# Liquid NanoBiosensors Enable One‐Pot Electrochemical Detection of Bacteria in Complex Matrices

**DOI:** 10.1002/advs.202207223

**Published:** 2023-04-23

**Authors:** Sara M. Imani, Enas Osman, Fatemeh Bakhshandeh, Shuwen Qian, Sadman Sakib, Michael MacDonald, Mark Gaskin, Igor Zhitomirsky, Deborah Yamamura, Yingfu Li, Tohid F. Didar, Leyla Soleymani

**Affiliations:** ^1^ School of Biomedical Engineering McMaster University 1280 Main Street West Hamilton ON L8S 4L7 Canada; ^2^ Department of Engineering Physics McMaster University 1280 Main Street West Hamilton ON L8S 4L7 Canada; ^3^ Department of Biochemistry and Biomedical Sciences McMaster University 1280 Main Street West Hamilton Ontario L8S 4L8 Canada; ^4^ Department of Materials Science and Engineering McMaster University 1280 Main Street West Hamilton Ontario L8S 4L8 Canada; ^5^ Hamilton Regional Laboratory Medicine Program Hamilton General Hospital 237 Barton St. East Hamilton Ontario L8L 2×2 Canada; ^6^ Department of Pathology and Molecular Medicine McMaster University 1280 Main Street West Hamilton Ontario L8S 4K1 Canada; ^7^ Department of Mechanical Engineering McMaster University Hamilton ON L8S 4L7 Canada; ^8^ Institute of Infectious Disease Research McMaster University Hamilton ON L8N 3Z5 Canada

**Keywords:** bacteria detection, DNAzymes, electrochemical biosensors, liquid‐infused surfaces, urinary tract infections

## Abstract

There is a need for point‐of‐care bacterial sensing and identification technologies that are rapid and simple to operate. Technologies that do not rely on growth cultures, nucleic acid amplification, step‐wise reagent addition, and complex sample processing are the key for meeting this need. Herein, multiple materials technologies are integrated for overcoming the obstacles in creating rapid and one‐pot bacterial sensing platforms. Liquid‐infused nanoelectrodes are developed for reducing nonspecific binding on the transducer surface; bacterium‐specific RNA‐cleaving DNAzymes are used for bacterial identification; and redox DNA barcodes embedded into DNAzymes are used for binding‐induced electrochemical signal transduction. The resultant single‐step and one‐pot assay demonstrates a limit‐of‐detection of 10^2^ CFU mL^−1^, with high specificity in identifying *Escherichia coli* amongst other Gram positive and negative bacteria including *Klebsiella pneumoniae*, *Staphylococcus aureus*, and *Bacillus subtilis*. Additionally, this assay is evaluated for analyzing 31 clinically obtained urine samples, demonstrating a clinical sensitivity of 100% and specify of 100%. When challenging this assay with nine clinical blood cultures, *E. coli*‐positive and *E. coli*‐negative samples can be distinguished with a probability of *p* < 0.001.

## Graphical abstract

Nonspecific binding of undesired species poses a challenge for biosensing in complex media. Herein, a repellent liquid‐infused nanostructured biosensor is developed, enabling the specific detection of *Escherichia coli* in complex physiological media in a one‐pot assay within an hour.

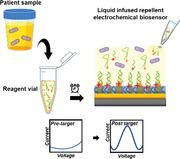

## 1 Introduction

Electrochemical biosensors that use nucleic acids as the biorecognition element have been developed for the detection and identification of bacteria.^[^ ^1^, ^2^ ^]^ These systems provide a rapid and point‐of‐care alternative to growth culture‐based techniques for infectious disease diagnostics and are envisioned to be less operationally‐complex and expensive than methods using nucleic acid amplification.^[^ ^1^, ^3^, ^4^ ^]^ Despite great promise, these electrochemical systems often lack simplicity due to the need for 1) sample processing when working with clinical specimens ^[^ ^3^, ^4^ ^]^ and 2) step‐wise addition of reagents inhibiting one‐pot and one‐step operation.^[^ ^5^, ^6^, ^7^ ^]^ One key reason for the two abovementioned challenges is nonspecific binding.^[^ ^8^ ^]^ First, nonspecific binding reduces the signal generated on the biosensor surface, requiring sample processing steps such as target extraction and specimen dilution. Second, the presence of signal‐generating probes such as redox molecules can generate large background signals due to nonspecific interaction of these molecules with the sensor surface in the absence of the target.^[^ ^8^ ^]^ To overcome these limitations, nucleic acid probes on the electrode surface are commonly mixed with self‐assembled monolayers such as mercapto hexanol, hexane dithiol, dithiothreitol, and ethylene glycols.^[^ ^9^, ^10^, ^11^ ^]^ These methods are generally effective but still require specimen dilution when working with complex matrices, which adds to the operational complexity of assays and reduces the amount of target molecules available for analysis.^[^ ^12^, ^13^, ^14^ ^]^ Other blocking agents commonly used in optical immunoassays such as bovine serum albumin are also used in electrochemical assays^[^ ^15^ ^]^; however, these result in the passivation of the electrode surface and the reduction of conductivity. Strategies for preserving electrode conductivity while reducing nonspecific binding have been developed by creating networks of antifouling reagents and conductive nanoparticles, ^[^ ^16^ ^]^ as well as developing anti‐biofouling conductive polymers.^[^ ^17^, ^18^ ^]^ Liquid‐infused surfaces provide an alternative for creating anti‐biofouling electrochemical biosensors since they provide repellency against a broad range of background materials ^[^ ^19^, ^20^, ^21^, ^22^, ^23^ ^]^ while preserving electrochemical activity of surfaces.^[^ ^24^ ^]^ These surfaces have shown strong repellency against complex media such as blood and plasma, ^[^ ^21^, ^25^, ^26^ ^]^ pathogenic contamination, ^[^ ^26^, ^27^, ^28^ ^]^ as well as proteins, ^[^ ^25^, ^29^ ^]^ which has not been collectively achieved using other anti‐biofouling strategies.^[^ ^20^, ^25^ ^]^ This makes liquid‐infused surfaces ideally suited for one‐pot biosensing in which a heterogeneous mixture of known and unknown background materials is present.

Herein, we sought to develop a one‐pot bacterial sensor by combining liquid‐infused nanostructured electrodes (Liquid NanoBiosensors) with redox DNAzymes for one‐pot bacterial sensing. We studied whether it would be possible to mix clinical specimens with redox DNAzymes and use this solution for one‐pot sensing. We compared the ability of Liquid NanoBiosensors with standard nanostructured electrodes (NanoBiosensors) in supressing the background signals generated by redox DNAzymes and reducing biofouling in blood, plasma, and urine, and benchmarked their limit‐of‐detection. We finally evaluated the ability of the one‐pot assay in identifying *Escherichia coli* in specimens from patients with urinary tract and bloodstream infections.

## 2 Results and Discussion

### 2.1 Fabricating Liquid NanoBiosensors

**Figure 1:**
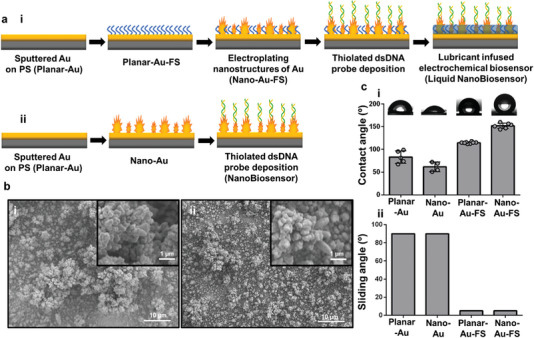
Fabrication and characterization of Liquid NanoBiosensors. a) Steps for creating i) Liquid NanoBiosensors and ii) NanoBiosensors. b) Scanning electron micrographs of i) Nano‐Au‐FS and ii) Nano‐Au surfaces. c) Contact i) and sliding angle ii) measurements of Planar‐Au, Nano‐Au, Planar‐Au‐FS, and Nano‐Au‐FS surfaces by depositing a 5 µL deionized water droplet. For contact angle measurements, no lubricant was added, and for sliding angle measurements, a lubricating layer was perfused. For each measurement, at least four separate samples were used. Representative images of the contact angle of water droplets are shown in (i).

In order to fabricate Liquid NanoBiosensors, 100 nm of Au was sputtered on polystyrene surfaces (Planar‐Au), which were covered by a mask containing a star‐shaped electrode design (**Figure** **1**a). After mask removal, the electrodes were fluorosilane (FS) treated using chemical vapor deposition method (Planar‐Au‐FS) to lock down a fluorocarbon liquid layer for creating a repellent coating. After chemical vapor deposition, nanostructures of gold were created by electroplating (Nano‐Au‐FS) as shown in the scanning electron microscopy (SEM) images in Figure 1b(i). The star‐shaped design, having multiple sharp edges, leads to diffusion‐limited growth and high‐aspect‐ratio electrodeposited architectures, providing a high surface area for thiolated DNA probes to bond to the electrode surface via thiol–gold chemistry (Figure 1b; Figure S1, Supporting Information).^[^ ^3^, ^30^ ^]^ Gold electrodes where also electroplated without any prior FS treatments (Nano‐Au) to provide a control group against Nano‐Au‐FS. Similar to Nano‐Au‐FS surfaces, Nano‐Au electrodes demonstrated nanostructured features (Figure 1b(ii); Figure S1a, Supporting Information); however, the structures found on the latter are smaller. The larger features on Nano‐Au‐FS electrodes can be attributed to the negatively charged FS‐covered surface, attracting more positively charged Au ions from the electroplating solution, leading to electroplating larger structures per each growth point. The electroactive surface area was also evaluated (Figure S2a–c, Supporting Information), showing similar redox characteristics for Nano‐Au‐FS and Nano‐Au electrodes, however, Nano‐Au showed 28% less electroactive surface area than Nano‐Au‐FS. Potentiodynamic studies were performed for Nano‐Au, Nano‐Au‐FS, and L‐Nano‐Au‐FS (liquid‐infused Nano‐Au‐FS) and the testing results were presented in Tafel plots (Figure S3, Supporting Information). The samples showed low corrosion currents as these surfaces are comprised of a noble metal. The corrosion current of L‐Nano‐Au‐FS is an order of magnitude less then the other control groups which indicates liquid infusion of L‐Nano‐Au‐FS liquid surfaces and their associated anti‐corrosion behavior. This behavior is inline with studies from literature focusing on liquid‐infused surfaces having an anti‐corrosion effect.^[^ ^31^, ^32^ ^]^ The pitting potentials (Figure S4, Supporting Information) of Nano‐Au, Nano‐Au‐FS, and L‐Nano‐Au‐FS are in a similar range (0.69, 0.66, 0.69 V). This shows that the nanostructured Au can be exposed to the electrolyte even though the lubricating layer exists.^[^ ^33^ ^]^

Successful immobilization of the FS monolayer and repellent behavior of the surfaces were evaluated by contact angle and sliding angle measurements (Figure 1c(i,ii)). Planar‐Au and Nano‐Au surfaces showed a static water contact angle of 83 ± 12° and 61 ± 9°, which was then elevated to 115 ± 1° and 152 ± 5° after FS treatment for FS‐Au and Nano‐Au‐FS, respectively. This was due to the decrease in the surface free energy caused by the FS treatment, leading to a higher Cassie‐Baxter contact angle, demonstrating successful FS immobilization. The increase in contact angles from FS‐Au to Nano‐Au‐FS, is due to the presence of multiple structural length scales in Nano‐Au‐FS and having more stable Cassie‐Baxter states while having a low surface energy.^[^ ^34^, ^35^ ^]^ The decrease in contact angle from Au to Nano‐Au can be explained by the existence of the Wenzel state, in which the contact angle decreases for rough surfaces with a contact angle below 90°.^[^ ^34^, ^35^ ^]^ Furthermore, sliding angle measurements were performed after liquid infusion on both FS‐treated and non‐FS‐treated surfaces. The liquid‐infused FS‐treated surfaces (L‐Planar‐Au‐FS and L‐Nano‐Au‐FS) showed sliding angles below 5° as water droplets immediately started to move and slide off (shown as 5° on Figure 1c(ii)). On the non‐FS‐treated surfaces (L‐Planar‐Au and L‐Nano‐Au), the drops got pinned and did not move (shown as 90° on Figure 1c(ii)). The L‐Nano‐Au‐FS electrodes demonstrate a repellent surface that is expected to reduce interference by unwanted molecules on the surface of biosensors. As such, this surface was modified with DNA probes, creating an electrochemical biosensor (Liquid NanoBiosensor). The charge transfer characteristics of the three surfaces were characterized by Electrochemical Impedance Spectroscopy (EIS) in Figure S5 (Supporting Information). The data were analyzed using Randles circuit. The Nano‐Au, Nano‐Au‐FS, and L‐Nano‐Au‐FS showed charge transfer resistance (Rct) values of 8.8 ± 5.5, 33 ± 4.8, and 35.6 ± 8.4 Ω, respectively The increase in the Rct value of Nano‐Au‐FS, and L‐Nano‐Au‐FS compared to Nano‐Au is caused by chemical modification and lubricant infusion. In order to assess the anti‐biofouling behavior of the three surfaces using EIS, Nano‐Au, and L‐Nano‐Au‐FS were incubated in blood for 30 s and washed. The change in the Rct value was documented for the three surfaces following blood incubation. L‐Nano‐Au‐FS surfaces showed approximately four times less percentage change of the Rct value than Nano‐Au electrodes, which is attributed to the improved anti‐biofouling properties of L‐Nano‐Au‐FS electrodes.

### 2.2 Developing a One‐Pot Assay Using Liquid NanoBiosensors

**Figure 2:**
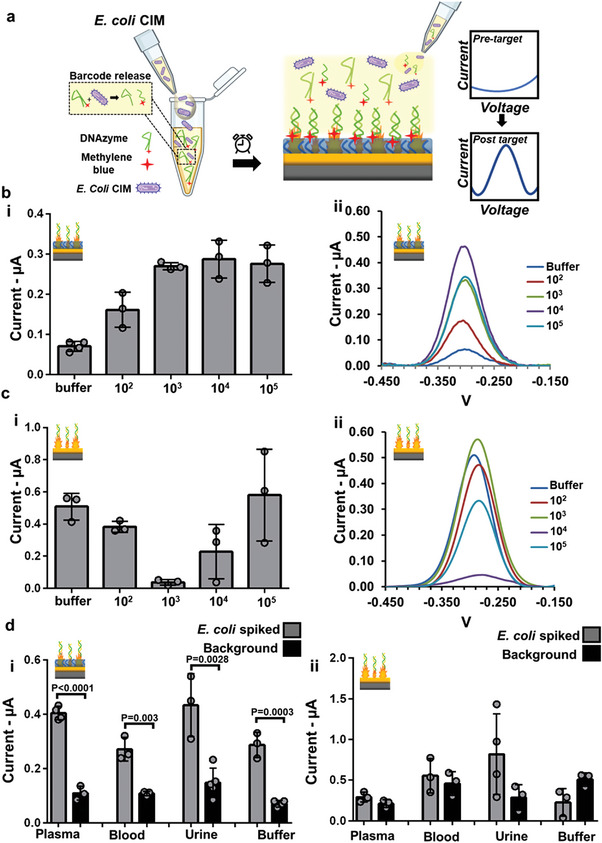
*E. coli* detection using Liquid NanoBiosensors and NanoBiosensors. a) Schematic diagram depicting the assay operation. *E. coli* CIM is added to the reaction vial containing DNAzymes, resulting in redox barcode release. Following incubation for 30 min at room temperature, a drop is positioned onto the electrochemical chip for 30 min for analysis. The capture of the redox barcode by the probe‐modified electrodes results in an increase in the measured electrochemical current. Bar plots (i) and representative square wave voltammograms (ii) measured in buffer solutions containing different concentrations of *E. coli* measured using Liquid NanoBiosensors (b) and NanoBiosensors (c). d) Analysis of *E. coli* at a concentration of 10^4^ CFU mL^−1^ spiked in plasma, blood, urine, and buffer.

In order to create a one‐pot electrochemical assay for detecting Escherichia coli, we used RNA‐cleaving DNAzymes tagged with electroactive species, methylene blue (tagging and purification steps discussed in Experimental Section), and included these along with all the necessary assay reagents in a vial, in which the sample is added to later. The interaction between *E. coli* and methylene blue‐tagged DNAzyme results in the release of a DNA barcode (**Figure** **2**a), which is captured by the probe immobilized on the electrode (Figures 1a and 2a).^[^ ^3^, ^36^ ^]^ Similar to the commercially‐available rapid tests, the assay involves two steps. First, the specimen is added to the reagent vial (30 min), and then a drop of the reagent/specimen mixture is added to the biosensor (30 min), after which the electrochemical current is measured. The reagent vial contains DNAzymes, whereas the specimen consists of *E. coli* spiked in buffer, biological fluids, or clinical samples derived from individuals with an *E. coli* infection. In this one‐pot assay, the DNAzymes in the reagent vial release redox barcodes upon reaction with *E. coli*. Once the reagent mixture containing *E. coli* is introduced to the chip, the released redox barcodes are captured by the probe‐modified electrodes, demonstrating an increase in the measured signal (Figure 2a). The thiolated DNA probes used herein are double‐stranded, one strand is complementary to the released DNA barcode, while the other strand serves as a protector to reduce the probability of hybridization between the thiolated probe and the unreacted DNAzyme. Our results indicate that the use of this double‐stranded probe is critical in ensuring a sufficient target‐to‐blank ratio (Figure S6, Supporting Information). Immobilization of the double‐stranded DNA probe was validated using cyclic voltammetry on both Nano‐Au‐FS and Nano‐Au electrodes (Figure S2d,e, Supporting Information), showing probe deposition on both classes of electrodes. The modification of these electrodes with probe DNA allows us to construct Liquid NanoBiosensors and NanoBiosensors (control surface). It should be noted that both Nano‐Au‐FS and Nano‐Au electrodes showed well‐defined redox peaks with similar current magnitudes (5.6 µA for Nano‐Au‐FS and 5.05 µA for Nano‐Au) and peak‐to‐peak separation (0.064 V for Nano‐Au‐FS and 0.068 V for Nano‐Au; Figure S2d,e, Supporting Information), making the Liquid NanoBiosensors a viable electrode system for electrochemical signal readout.

To evaluate the performance of the Liquid NanoBiosensors and compare them with NanoBiosensors, the two classes of electrodes were used to analyze the crude intracellular matrix (CIM) of *E. coli* at varying concentrations (10^2^–10^5^ CFU mL^−1^). Following the on‐chip incubation of bacteria, the solution was washed off and a negative pulse was applied to the electrodes to remove weakly bound or non‐specifically adsorbed DNA strands. This practice reduced the variability between measurements performed using different chips. (Figure S7, Supporting Information). The Liquid NanoBiosensors were able to detect *E. coli* at a concentration of 10^2^ CFU mL^−1^ in buffer (Figure 2b(i,ii)); whereas, the NanoBiosensors, showed indistinguishable data from the blank for all tested concentrations, leaving the detection inconclusive (Figure 2c(i,ii)). This shows that the repellent coating on the Liquid NanoBiosensors enhances the limit‐of‐detection of the assay by lowering the blank signal. The Liquid NanoBiosensors demonstrated a saturation in their response beyond 10^3^ CFU mL^−1^, indicating that the probes on these electrodes could be reaching their maximum hybridization efficiency at this concentration.^[^ ^3^, ^37^, ^38^ ^]^

We also investigated reusing the Liquid NanoBiosensors by regenerating them by scanning them in H_2_SO_4_. We compared the performance of the regenerated electrodes with new ones. As shown in Figure S8 and Note S1 (Supporting Information), there are no significant changes in the signal observed for the regenerated and new electrodes.

### 2.3 Evaluating Liquid NanoBiosensors Using Various Specimens and Control Bacteria

The ability of the assay in detecting *E. coli* in various complex biological liquids was also tested. Human plasma, whole blood, urine, and buffer were spiked with 10^4^ CFU mL^−1^ of *E. coli* and tested using NanoBiosensors and Liquid NanoBiosensors (Figure 2d(i,ii)). For all *E. coli* samples, the Liquid NanoBiosensors showed significantly higher signals (*p* ≤ 0.003) from the target solution compared to blanks. However, the NanoBiosensors demonstrated indistinguishable signals when comparing spiked specimens and blanks. This is likely due to the lower background signals on Liquid NanoBiosensors (<150 nA) compared to NanoBiosensors (211–508 nA). Based on these findings, we expected the Liquid NanoBiosensors to be suitable for one‐pot analysis of clinical specimens.

**Figure 3:**
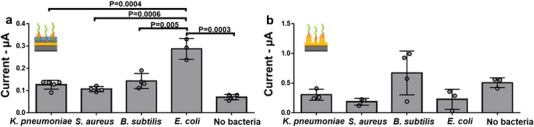
*E. coli* specificity test – Specificity test performed by mixing a 10^4^ CFU mL^−1^ concentration of *K. pneumoniae*, *S aureus*, *B. subtilis*, *E. coli*, or buffer with redox DNAzymes followed by incubation on (a) Liquid NanoBiosensors and (b) NanoBiosensors. The error bars represent the standard deviation from the mean obtained using at least three separate electrodes per sample.

To validate the specificity of the assay and its ability to identify *E. coli* among other bacteria, various Gram‐negative and Gram‐positive bacteria (*Klebsiella pneumoniae*, *Staphylococcus aureus*, *Bacillus subtilis*, and *E. coli* at 10^4^ CFU mL^−1^) were analyzed using Liquid NanoBiosensors and NanoBiosensors (**Figure** **3**a,b). The Liquid NanoBiosensors demonstrated stronger and statistically distinguishable electrochemical signal for *E. coli* compared to other bacteria. Such characteristics were not observed for NanoBiosensors, as they showed higher signals for *B. subtilis* and buffer compared to *E. coli* (Figure 3b). These data confirm that Liquid NanoBiosensors reduce interference from unwanted species in a complex specimen, leading to specificity in bacterial identification. The blank signal in the absence of bacteria is reduced by 86% for the Liquid NanoBiosensors compared to the NanoBiosensors, whereas the target signal in the presence of *E. coli* is increased by 21%. This increase in target‐to‐blank ratio is important in enabling us to specifically identify the target analyte.

### 2.4 Detecting Clinical Urinary Tract Infections (UTIs) Caused by *E. coli*

**Figure 4:**
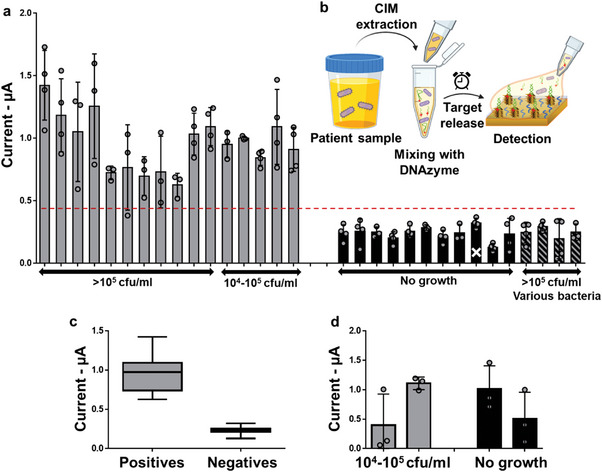
Analyzing patient urine samples. a) The Liquid NanoBiosensors evaluated using 11 *E. coli*+/culture+ (>10^5^ CFU mL^−1^), five *E. coli*+/culture+ (10^4^–10^5^ CFU mL^−1^), 11 *E. coli−*/culture−, and four *E. coli−*/culture+ (>10^5^ CFU mL^−1^ of *Enterococcus*, *K. oxytoca*, *S. aureus*, *K. pneumoniae*) samples tested using Liquid NanoBiosensors. b) Schematic illustration of patient urine analysis. c) Box and whisker plot demonstrating the distribution of *E. coli*+ and *E. coli*‐ samples analyzed in (a). d) Analysis of two *E. coli*+/culture+ (>10^5^) and two *E. coli*‐/culture‐ samples using NanoBiosensors.

Given the promising behavior of Liquid NanoBiosensors in analyzing spiked samples, we sought to determine whether this assay could analyze specimens from patients having symptoms of urinary tract infections. We collected 31 urine samples from such patients, which included 11 *E. coli*+/UTI+ (>10^5^ CFU mL^−1^), 5 *E. coli*+/UTI+ (10^4^ ‐10^5^ CFU mL^−1^), 11 *E. coli*‐/UTI‐, and 4 *E. coli*‐/UTI+ (>10^5^ CFU mL^−1^ of *Enterococcus*, *Klebsiella oxytoca*, *S. aureus*, *K. pneumoniae*) specimens (**Figure** **4**a). These clinical specimens were added to the reagent vial and were tested on Liquid NanoBiosensors (Figure 4b). To determine the clinical sensitivity and specificity of this assay, a detection threshold of 470 nA was calculated based on a 95% confidence interval by GraphPad Prism. This threshold led to a sensitivity of 100% and specificity of 100%, enabling categorization of *E. coli*+ samples from the *E. coli*‐ ones (Figure 4c). This was in accordance with > 10^4^ CFU mL^−1^ threshold for UTI+ patients.^[^ ^3^, ^39^, ^40^, ^41^ ^]^ To evaluate the diagnostic ability of the developed platform, A receiver operating characteristic (ROC) plot was constructed (Figure S10, Supporting Information). The area under the curve (AUC) was measured to be 1 for the ROC plot, which meets the standard criteria for evaluating diagnostic tests.

The NanoBiosensors were also tested (Figure 4d) for a smaller subset of clinical samples (2 *E. coli*+/culture+ (>10^5^ CFU mL^−1^) and 2 *E. coli−*/culture−). As expected, there were no substantial differences detected for the no growth and positive samples. Furthermore, similar to the above‐mentioned methodology, *E. coli* CIM was spiked in healthy urine samples (Figure S9, Supporting Information), showing successful detection for the Liquid NanoBiosensors in the 10^2^–10^5^ CFU mL^−1^ range.

### 2.5 Detecting Bloodstream Infections Caused by *E. coli*

**Figure 5:**
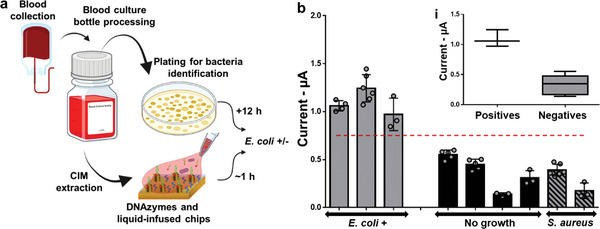
Analyzing patient blood samples. a) Schematic diagram demonstrating *E. coli* detection in the blood of symptomatic patients. b) Analysis of blood from patients with symptoms of bacteremia using Liquid NanoBiosensors. The inset demonstrates that the *E. coli‐*infected samples are statistically significant from the non‐infected ones.

To assess whether the one‐pot assay developed herein would be effective in diagnosing bloodstream infections, we first used Liquid NanoBiosensors to analyze *E. coli* spiked in healthy blood culture samples (10^2^ – 10^5^ CFU mL^−1^). As demonstrated in Figure S11 (Supporting Information), spiked samples showed distinguishable signals from the healthy blood sample at concentrations higher than 10^2^ CFU mL^−1^. Given the success of this experiment, we further analyzed specimens derived from patients suspected of having bloodstream infections or bacteremia (**Figure** **5**a). Clinical blood cultures having *E. coli* (quantified in Figure S12 and Note S2, Supporting Information), *S. aureus*, or no bacteria were acquired from patients with symptoms of bacteremia after the samples were analyzed using the liquid culture‐based BacT/ALERT system.^[^ ^42^ ^]^ The blood cultures were added to the reagent vial and subsequently incubated on Liquid NanoBiosensors (Figure 5b).

Among these specimens, three were *E. coli*+, two were *E. coli*‐/culture+ (*S. aureus+*), and four were culture−. The Liquid NanoBiosensors were able to specifically detect *E. coli*‐infected samples by demonstrating larger signal amplitudes than *S. aureus*‐infected and negative blood samples, differentiating *E. coli*+ samples from the other control groups (Figure 5a). Even though our analysis includes a limited number of clinical specimens, our ability in identifying the microorganism causing the bloodstream infection without the commonly used sub‐cultures conducted following the use of the BacT/Alert system ^[^ ^42^ ^]^ shows the promise of our approach in rapid bacterial identification. Rapid bacterial identification is critical for patients with bloodstream infections as it will guide physicians in prescribing precision and effective treatments more rapidly for a condition that is time sensitive with a high fatality rate.^[^ ^43^, ^44^ ^]^

## 3 Conclusions

Pathogen testing in complex and unprocessed clinical specimens using simple, culture‐free, and rapid assays is challenging due to interference of the sample matrix and the signaling probes with the biosensor. To develop a one‐pot electrochemical assay that is applicable to a range of specimen types, we created a new class of anti‐biofouling electrodes and combined these with *E. coli*‐specific redox DNAzymes that release a redox DNA barcode in response to the target. These released targets are detected on anti‐biofouling electrodes, which 1) reduce the interference of the unreacted redox DNAzymes with the electrode surface and as such reduce blank signals and 2) reduce the impact of the specimen interference on the analytical performance of the assay.

In this work, we identified *E. coli* in buffer, plasma, whole blood, and urine using the newly developed anti‐biofouling electrodes, which was not possible using electrodes that lacked this surface treatment. Clinical specimens from symptomatic urinary tract infection patients were analyzed with a sensitivity of 100% and specificity of 100%. Additionally, specimens from patients with bloodstream bacteria were studied, and our assay was able to successfully identify the blood specimens containing *E. coli*. The one‐pot assay developed herein, enabled by the anti‐biofouling electrodes, can be easily applied to the detection of other pathogens, as well as other clinical biomarkers.

## 4 Experimental Section

### Materials

Trichloro(1H,1H,2H,2H‐perfluorooctyl)silane (FS), perfluoroperhydrophenanthrene (PFPP), 6‐mercapto‐1‐hexanol (MCH, 99%), tris(2‐carboxyethyl)phosphine hydrochloride (TCEP), sodium chloride (NaCl, ≥99.0%), magnesium chloride (MgCl_2_, ≥99.0%), phosphate buffer solution (1.0 m, pH 7.4), gold(III) chloride solution (HAuCl4, 99.99%), tris, ethylenediaminetetraacetic acid (EDTA), sodium acetate, HEPES buffer, bicarbonate buffer solution, and potassium hexacyanoferrate(II) trihydrate ([Fe(CN)6]4−, ≥99.95%) were purchased from Sigma–Aldrich. Hydrochloric acid (HCl; 37% w/w) was purchased from LabChem. Sulfuric acid (H_2_SO_4_, 98%) and isopropyl alcohol (IPA) (99.5%) were purchased from Caledon Laboratories. Methylene blue NHS ester was purchased from Glen Research (Virginia, USA). Urea and 40% 29:1 bis/acrylamide were purchased from Bioshop (Ontario, Canada). The DNA oligonucleotides were purchased from Integrated DNA Technologies (IDT) and Yale University. Sequence details can be found in Table S1 (Supporting Information). The water used in all experiments was purified with a Milli‐Q Synthesis A10 water‐purification system and further autoclaved.

### Electrode Fabrication

Polystyrene sheets (Graphix Shrink Film, Graphix) were cleaned with IPA and water and air‐dried. Subsequently, the sheet was covered with a vinyl mask (FDC 4304, FDC Graphic Films) and then cut into the working electrode pattern using a Robo Pro CE5000‐40‐CRP cutter (Graphtec America). The masked substrates were then sputtered with gold to a final thickness of 100 nm with a direct current sputtering machine (MagSput, Torr International). The sputtered working electrodes were then cut and cleaned with IPA. For fabricating Planar‐Au‐FS, the electrodes were placed in an air plasma cleaner (Harrick Plasma Cleaner, PDC‐002, 230 V), and exposed to high‐pressure air plasma for 3 min to be functionalized with hydroxyl groups. Then, they were transferred to a desiccator and 300 µL of FS was added in a container along with the electrodes in a separate container. The vacuum pump was then turned on until a pressure of −0.08 MPa was reached to start the chemical vapor deposition process to create self‐assembled monolayers or FS. The reaction was carried out for three hours at room temperature. Subsequently, the electrodes were removed from the desiccator and placed in an oven at 60 °C for a minimum of 18 h. To ensure the removal of non‐covalently attached FS molecules, the electrodes were sonicated for 5 min (VWR SympHony 97043–936 ultrasonic cleaner). Subsequently gold nanostructures were electrodeposited onto the electrodes by applying a static potential of −0.6 V for 600 s in a solution of 10 mm HAuCl_4_ and 5 mm HCl using a PalmSens4 (PalmSens) potentiostat with Ag/AgCl as the reference and platinum wire as the counter electrode to fabricate Nano‐Au‐FS.

### Methylene Blue Tagging and Purification

The lyophilized 5’‐Amino‐ modified *E. coli* DNAzyme was diluted in 0.1 m carbonate/bicarbonate buffer (pH 9) and mixed with methylene blue NHS Ester, then left to react for 2 h at room temperature following the manufacturer instructions. To remove the excess methylene blue, the DNAzyme is then purified using 10% urea 40% 29:1 Bis/Acrylamide page gel (denaturing gel). An Ethanol precipitation step was performed by adding 0.1× sodium acetate (pH 5.2), 2.5 × 100% ethanol and left at −20 °C for 2 h and centrifuged at 15 000 rpm, 4 °C. The precipitated sample was diluted in autoclaved water and loading dye, then loaded to the gel and run for 1 h (36 W). The DNAzyme bands were visualized using UV light (240 nm) and cut out. Afterward, the gel was crushed and eluted using an in‐house elusion buffer (200 mm NaCl, 10 mm Tris pH 7.5, 1 mm EDTA). The crushed gel was eluted two more times on a vortex at setting 3 for 30 min. A final ethanol precipitation step was applied, the retrieved DNAzyme was then diluted in RNA/DNA‐free water and stored in −20 °C for future use.

### CIM Preparation

The crude intracellular matrix (CIM) of *E. coli, S. aureus, K. pneumoniae*, and *B. subtilis* was prepared by culturing a single colony in LB media overnight until optical density OD600 of 1.0 (≈2 Å, ≈109 cells mL^−1^) was reached. After that, 1 mL of each bacterial culture was centrifuged at 10 000 × *g* for 10 min and the clear supernatant was discarded. Cells were then suspended in 500 µL of 1× reaction buffer (HEPES 50 mm, NaCl 150 mm, MgCl_2_ 15 mm, Tween 20 0.01%, pH 7.5). Subsequently, the cell suspension was lysed by heat (90 °C for 10 min). The Lysed cells were then centrifuged at 13 000 × *g* for 10 min and the clear supernatant was collected. Finally, the supernatant was passed through a 0.2 µm filter disc, aliquoted, and stored at −20 °C for future DNAzyme cleavage experiments. This CIM preparation protocol was adapted from Ref. ^[^ ^45^ ^]^z.

### Surface Physical Characterization

SEM imaging was performed using a JEOL 7000F. Transmission electron microscopy (TEM) imaging was performed using Thermo Scientific Talos 200× on the cross‐section of the electrodes prepared by an ultramicrotome. Briefly, electrodes were placed in flat embedding molds, then filled with epoxy resin and polymerized overnight at 60 °C.  From the blocks, thin sections were cut with a diamond knife on a Leica UCT Ultramicrotome and picked up onto Cu grids. Sections on grids were carbon‐coated prior to viewing in the TEM. The TEM was operated at 200 kV. Contact angle measurements were done using a goniometer (DSA30, Krüss Scientific, Hamburg, Germany) with water droplets (5 µL) dispensed by an automated syringe. The sessile drop contact angle was provided via image processing software (Krüss ADVANCE). Sliding angle measurements were done using a digital angle level (ROK, Exeter, UK) and prior to each measurement, the electrodes were infused with PFPP and a 5 µL water droplet was deposited. The angle at which point the droplet started to move was recorded as the sliding angle. Each mean value represented an average over at least three measurements.

### Probe Immobilization

After washing with IPE, the electrodes were electrochemically cleaned by applying cyclic voltammetry in 0.1 m H_2_SO_4_ (0–1.5 V, 100 mV s–1, 40 cycles). The electrodes were then incubated for 20 h with a 3 µL drop of 2 µm thiolated double‐stranded probe solution, which was reduced for 2 h prior to deposition with 2 mm TCEP solution in the dark at room temperature. After probe deposition, the electrodes were backfilled with 100 mm MCH for 15 min in the dark at room temperature. All of the electrochemical measurements were performed using a PalmSens4 (PalmSens).

### Electrochemical Characterization

The immobilization of the thiolated double‐stranded DNA probe was confirmed using a cyclic voltammetry scan from 0 V to 0.5 V at a scan rate of 50 mV s^−1^ in 2 mm potassium hexacyanoferrate(II) solution. Electroactive surface area measurements were done by scanning electrodes in 0.1 m H_2_SO_4_ using cyclic voltammetry (0–1.5 V, 100 mV s^−1^) by integrating the area under the reduction peak as explained in Figure S1 (Supporting Information). The Tafel test was performed using the BioLogic VMP‐300 Multichannel Potentiostat in 3% NaCl (w/v) aqueous solution with a 3‐electrode corrosion cell containing a working electrode, counter‐electrode (Pt mesh), and a reference electrode (saturated calomel electrode). The results of potentiodynamic studies (1 mV s^−1^ rate) are presented in Tafel plots and the corrosion potential and current are reported for Nano‐Au, Nano‐Au‐FS, and L‐Nano‐Au‐FS. Linear sweep voltammetry (LSV) studies were performed using a PalmSens4 (PalmSens) potentiostat on Nano‐Au, Nano‐Au‐FS, and L‐Nano‐Au‐FS by sweeping the potential between −1 and 1 V with respect to Ag/AgCl with a scan rate of 0.01 V s^−1^ in a 3% NaCl (w/v) aqueous solution. The pitting potential was calculated as the potential at which a rapid increase in the current is observed on the LSV curves, which is indicative of the tendency of the metal breakdown.^[^ ^46^, ^47^ ^]^ Electrochemical Impedance Spectroscopy (EIS) was performed using a PalmSens4 (PalmSens) potentiostat on Nano‐Au, Nano‐Au‐FS, and L‐Nano‐Au‐FS. EIS was performed using 2 mm [Fe(CN)_6_]^3‐/4−^ in 0.05 m KCl and 0.01 m PBS solution at open circuit potential versus Ag/AgCl reference electrode over a frequency range from 100 kHz to 0.1 Hz. The percentage change in Rct values before and after incubating electrodes in healthy blood culture and subsequent washing with water is reported.

### Detection of Bacteria in Buffer

The bacterial CIM samples were spiked in 25:25 buffer (25 mm PBS and 25 mm NaCl buffer) and mixed with methylene blue tagged DNAzyme (0.5 µm) in a 1:1 ratio and incubated in the dark at room temperature for 30 min. The FS‐treated electrodes (Nano‐Au‐FS) were infused with the lubricant (PFPP), then both Liquid NanoBiosensors and NanoBiosensors were incubated with 3 µL of the *E. coli* CIM and methylene blue tagged DNAzyme mixture for 30 min at 37 °C. Healthy urine samples were used instead of 25:25 buffer in the spiked experiments. *E. coli* concentrations of 10^5^ to 10^2^ CFU mL^−1^ were evaluated in both matrixes. To assess the specificity, three control CIMs of other bacteria (*S. aureus, K. pneumoniae, B. Subtilis*) at 10^4^ CFU mL^−1^ were measured and compared with the same concentration of *E. coli* and buffer. To assess the assay performance in complex matrices, whole blood, plasma, and unfiltered urine samples were spiked with 10^4^ CFU mL^−1^ of *E. coli* CIM.

### Clinical Evaluation Using Patient Urine Samples and Blood Culture Samples

Patient urine sample set (total 31) comprising 11 *E. coli*+/culture+ (>10^5^ CFU mL^−1^), 5 *E. coli*+/culture+ (10^4^ – 10^5^ CFU mL^−1^), and 11 *E. coli*‐/culture‐, and 4 *E. coli*‐/culture+ (>10^5^ CFU mL^−1^ of *Enterococcus*, *K. oxytoca*, *S. aureus*, or *K. pneumoniae*) were tested on Liquid NanoBiosensors. two *E. coli*+/culture+ (>10^5^ CFU mL^−1^) and two *E. coli*‐/culture‐ samples were tested on NanoBiosensors. For the assessment, CIM extraction was done such that urine samples were heat treated at 55 °C for 15 min followed by centrifugation at 11 000 × *g* for 5 min. The supernatant was then removed and mixed with methylene blue tagged DNAzyme in a 1:1 ratio and incubated in the dark at room temperature for 30 minutes. Subsequently, the mixture was incubated on the respected electrodes and electrochemical measurements were performed.

Patient blood samples composed of three *E. coli*+, two *S. aureus*+, and four negative samples. To acquire the samples, blood specimens were collected from symptomatic patients and transferred to blood culture bottles. When flagged positive, blood cultures were further sub‐cultured to verify the bacteria species. Vials of blood cultures (*E. coli*+, *S. aureus*+, and negatives) were transferred from Hamilton General Hospital to our lab.

## Conflict of Interest

The authors declare no conflict of interest.

## Author Contributions

T.F.D., L.S., and Y.L. designed and conceived the research. The experiments were designed and performed by S.M.I. E.O. performed oligo modification and preparation, F.B. performed bacterial sample preparation and performed the EIS analysis. S.Q. and Y.L. helped with DNAzyme design and preparation, as well as bacterial sample preparation. M.G. and D.Y. prepared and characterized clinical samples. S.S. and M.M. performed the Tafel test and analysis. I.Z. performed EIS, Tafel test, and LSV analysis and helped with the preparation of the revised manuscript. All authors worked on manuscript writing and revisions.

## Supporting information

Supporting Information

## Data Availability

The data that support the findings of this study are available from the corresponding author upon reasonable request.
