# Supplementary material for: Liquid NanoBiosensors Enable One‐Pot Electrochemical Detection of Bacteria in Complex Matrices
Source: Adv Sci (Weinh). 2023 Apr 23;10(19):2207223. doi: 10.1002/advs.202207223 (PMC10323635; doi:10.1002/advs.202207223)
Supplement: Supplementary file 1 — Supporting Information [file ADVS-10-2207223-s001.pdf]

ADVANCED SCIENCE
Open Access

Supporting Information

for *Adv. Sci.*, DOI 10.1002/advs.202207223

Liquid NanoBiosensors Enable One-Pot Electrochemical Detection of Bacteria in Complex Matrices

Sara M. Imani, Enas Osman, Fatemeh Bakhshandeh, Shuwen Qian, Sadman Sakib, Michael MacDonald, Mark Gaskin, Igor Zhitomirsky, Deborah Yamamura, Yingfu Li, Tohid F. Didar and Leyla Soleymani**

WILEY-VCH

Supplementary information

for

Liquid NanoBiosensors Enable One-pot Electrochemical Detection of Bacteria in Complex Matrices

Sara M. Imani [a], Enas Osman [a], Fatemeh Bakhshandeh[b], Shuwen Qian [c], Sadman Sakib, Michael MacDonald, Mark Gaskin, Igor Zhitomirsky, Deborah Yamamura, Yingfu Li, Tohid F. Didar, Leyla Soleymani**

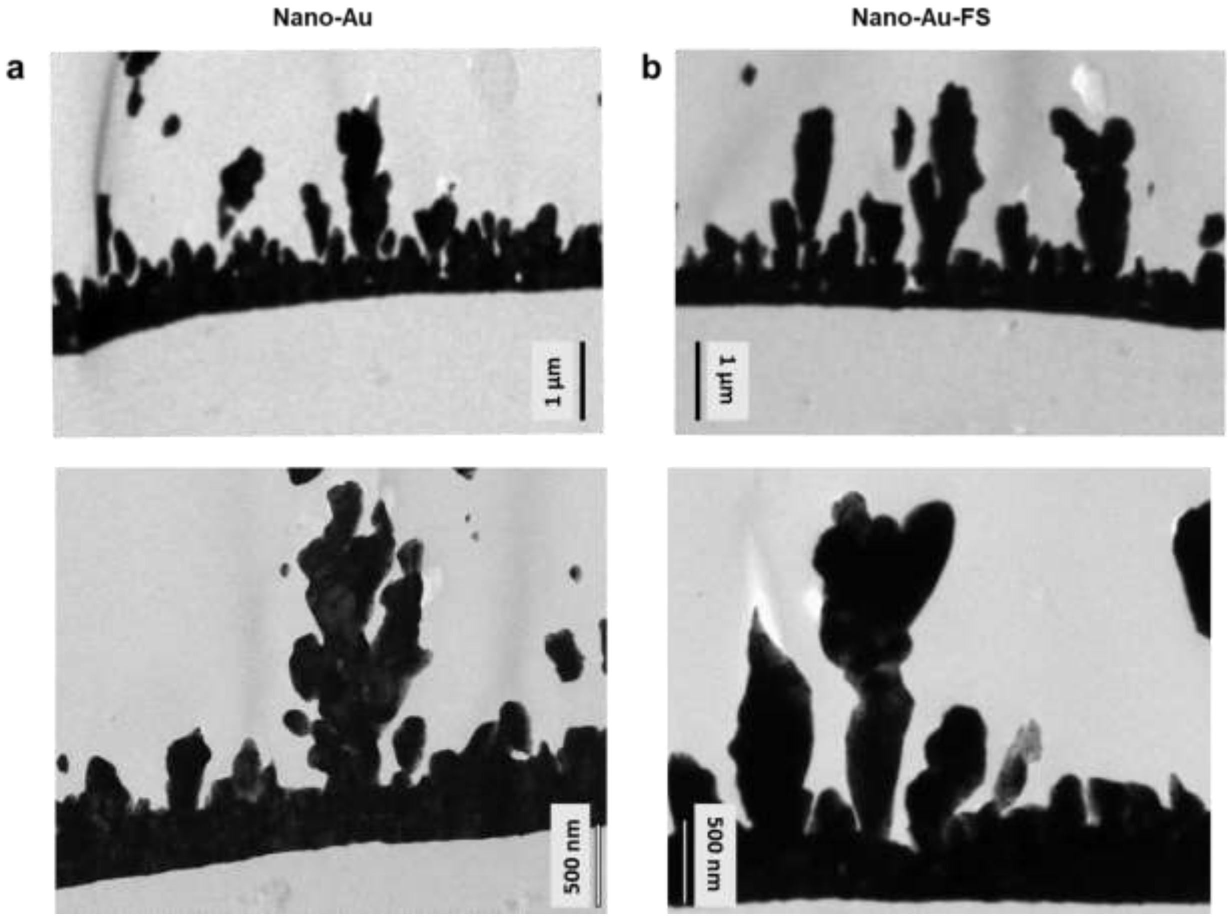

"Figure S1 – Transmission electron microscopy images of the cross section of a) Nano-Au and b) Nano-Au-FS surfaces, demonstrating the structure of the electroplated gold. Top images are zoomed out images (1µm scale bar) and bottom images are zoomed in images (500 nm scale bar)."

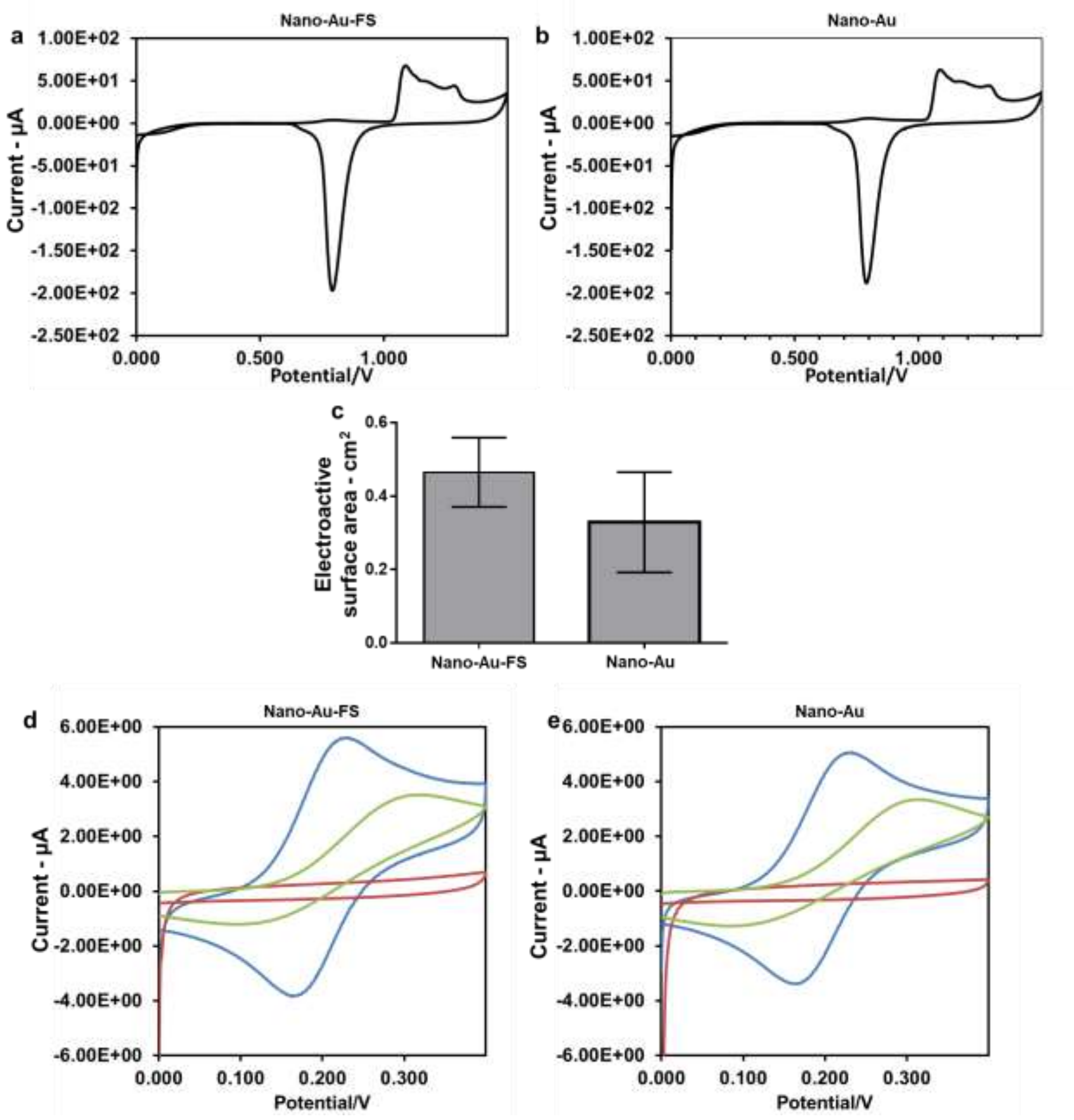

Figure S2 - Surface area characterization of (a) Nano-Au-FS and (b) Nano-Au electrodes in 0.1 M H_2SO_4 using cyclic voltammetry (0 V-1.5 V, 100 mV/s). (c) Electroactive surface area comparison. The electroactive surface area of the electrodes was calculated by performing a cyclic voltammetry (CV) scan from 0 to 1.5 V (against Ag/AgCl) in 0.1 M H_2SO_4 using a scan rate of 0.1 V/s. The area under the reduction peak was integrated and substituted in $\Gamma = A / (v*386\ \mu C\ cm^{-2})$, where Γ is the electrochemical surface area, A is the reduction peak area in the cyclic voltammogram and v is the scan rate. [1,2] Validation of probe deposition for Nano-Au-FS

(d) and Nano-Au (e) electrodes *via* cyclic voltammetry (0 V-0.5 V, 100 mV/s) in 2 mM potassium hexacyanoferrate (II) before probe deposition (blue), after probe deposition (red), and after backfilling with mercapto-1-hexanol (green).

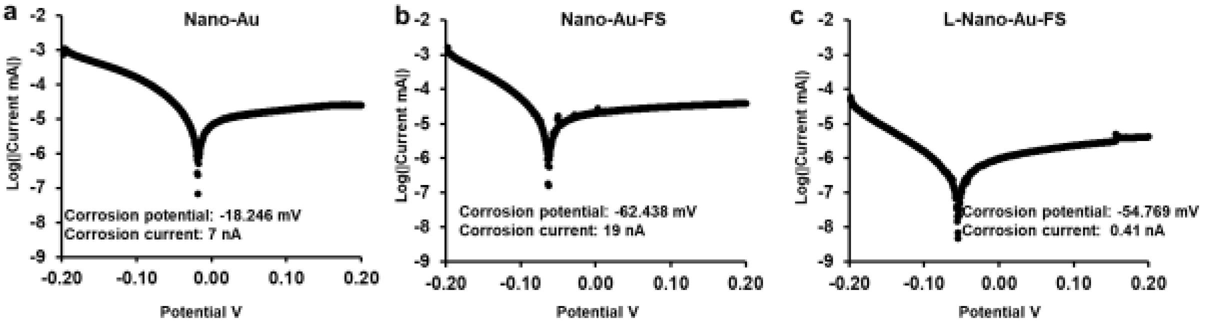

Figure S3 – Tafel plots for (a) Nano-Au, (b) Nano-Au-FS, and (c) L-Nano-Au-FS

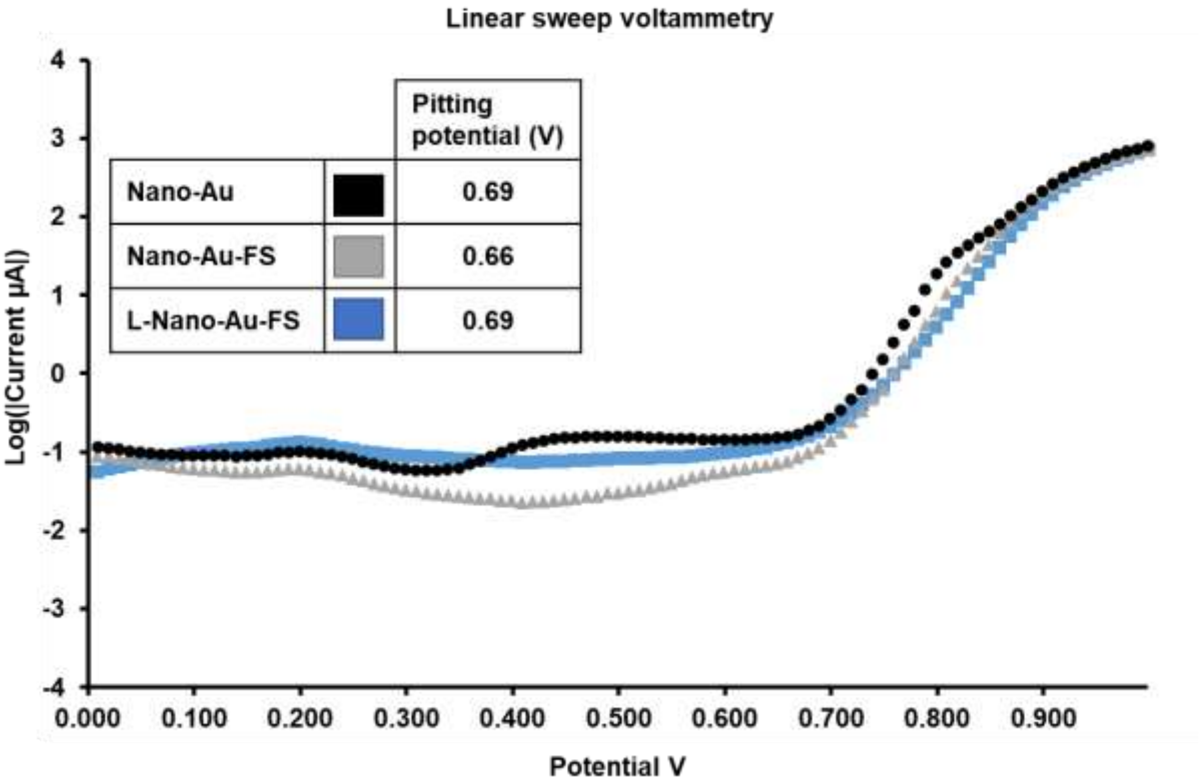

Figure S4 – LSV and pitting potentials for Nano-Au, Nano-Au-FS, and L-Nano-Au-FS.

a

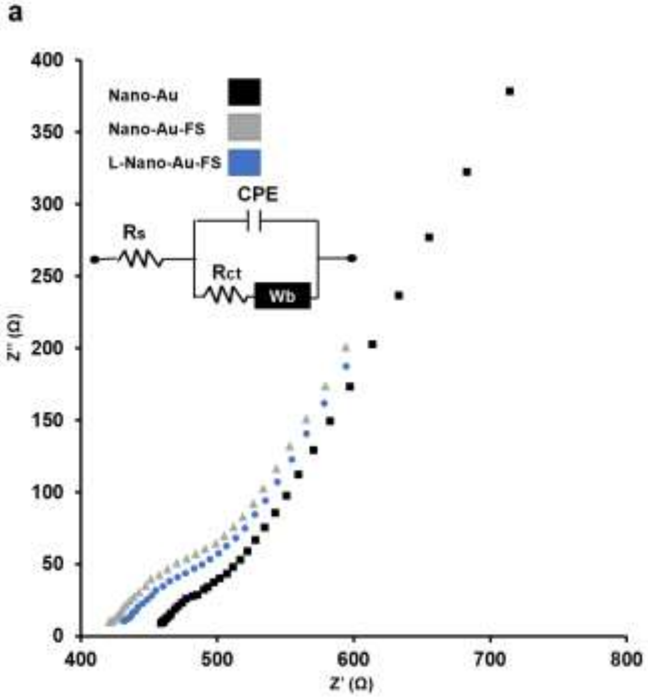

b

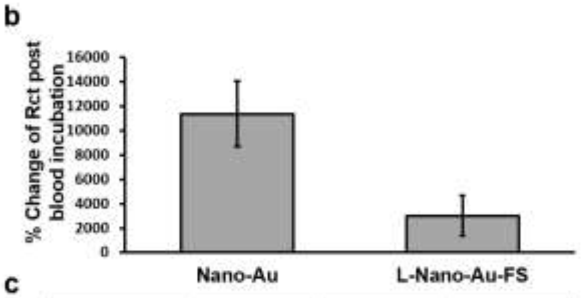

c

Sample	R_{ct} (Ω)	R_s (Ω)	C_{PE} (nF)
Nano-Au	13	469	5265
Nano-Au	12.4	447	650
Nano-Au	1	455	2250
Nano-Au-FS	39.3	430	1391
Nano-Au-FS	28	428	1741
Nano-Au-FS	30.6	419	1622
L-Nano-Au-FS	34.9	479	1428
L-Nano-Au-FS	25.7	428	1152
L-Nano-Au-FS	46.2	494	1713
Post blood incubation			
Nano-Au	725	426	2605
Nano-Au	1000	477	2734
Nano-Au	1300	462	2350
L-Nano-Au-FS	1243	485	3349
L-Nano-Au-FS	1748	586	2503
L-Nano-Au-FS	349	470	2523

Figure S5 – (a) EIS measurements of Nano-Au, Nano-Au-FS, and L-Nano-Au-FS. **(b)** Percentage change of **Rct for** Nano-Au and L-Nano-Au-FS after incubation in blood. (c) fitted parameters for each electrode.

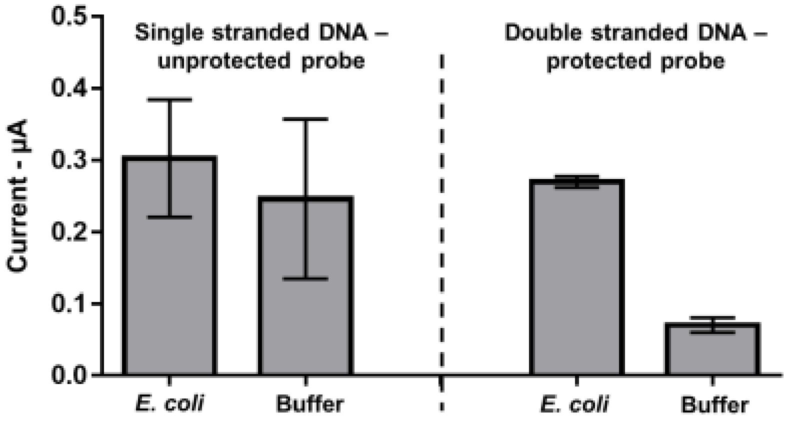

Figure S6 – Comparing single stranded DNA probe (unprotected probe) *vs.* double stranded DNA probe (protected probe) for detecting *E. coli* (10^3 CFU/mL) in buffer. The results indicate that the protected strand results in a larger target-to-blank ratio (increase from 1.2 to 3.9) as well

as less variation for each sample compared to the non-protected strand. The electrodes used in this study are Liquid NanoBiosensors.

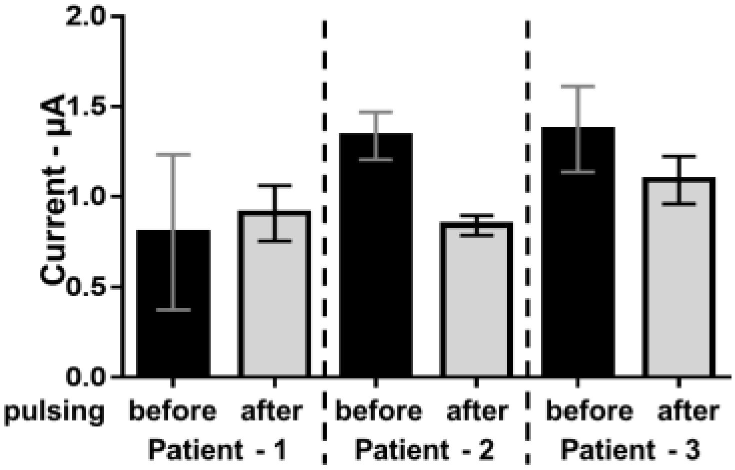

Figure S7 – To reduce variability between measurements performed using different chips, a negative pulse was applied to the electrodes to remove weakly bound or non-specifically adsorbed DNA strands. The signal from three difference *E. coli* positive urine samples were measured before and after applying the pulse. For each column at least three different electrodes were used. The results indicate reduction in data variability by applying the pulse.

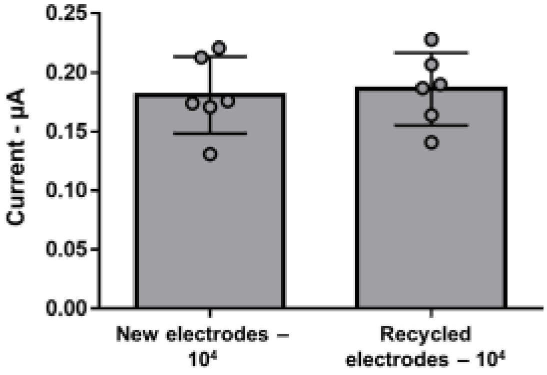

Figure S8 - The electrochemical current measurement after a solution of 10^4 CFU/mL of *E. coli* CIM was spiked in buffer and tested on new and recycled electrodes

Note S1 – After testing the electrodes with spiked *E. coli* samples, they were subjected to 0.1 M H_2SO_4 and scanned using cyclic voltammetry (0 V-1.5 V, 100 mV/s) to remove any bound

molecules to the surface (DNA probe, DNAzyme). Subsequently, the steps involved in the assay development were repeated (e.g. probe deposition, target capture, *etc.*). As showed in Figure S4, there are no significant changes observed in the signal. This finding demonstrates that our electrodes can be recycled for subsequent use.

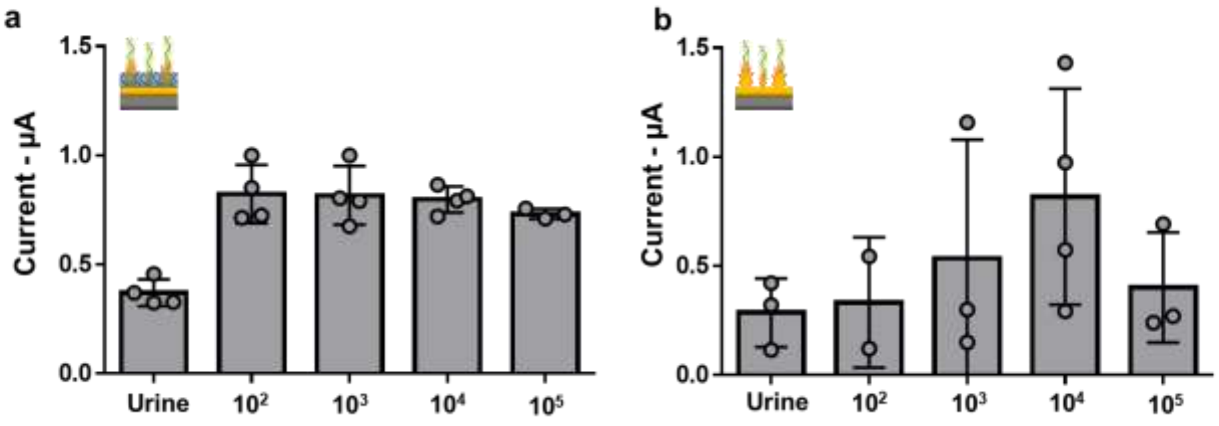

Figure S9 - Evaluating the limit-of-detection of Liquid NanoBiosensors and NanoBiosensors for detecting *E. coli* spiked in healthy human urine (a). Varying concentrations of *E. coli* CIM was spiked in urine and added to the reagent vial for 30 minutes at room temperature. The spiked urine sample was diluted by 50% during sample processing. A 3 µL drop of the solution was then added to the chip for 30 min at 37 °C. (b) The NanoBiosensors electrodes were evaluated similarly to the Liquid NanoBiosensors.

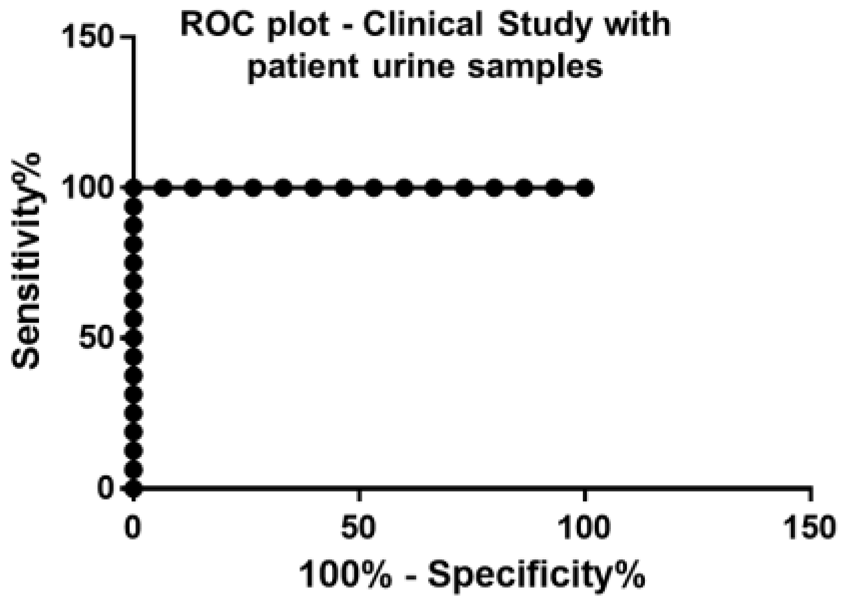

Figure S10 – Receiving operating characteristic (ROC) curve obtained for the clinical data presented in Figure 5 of the manuscript.

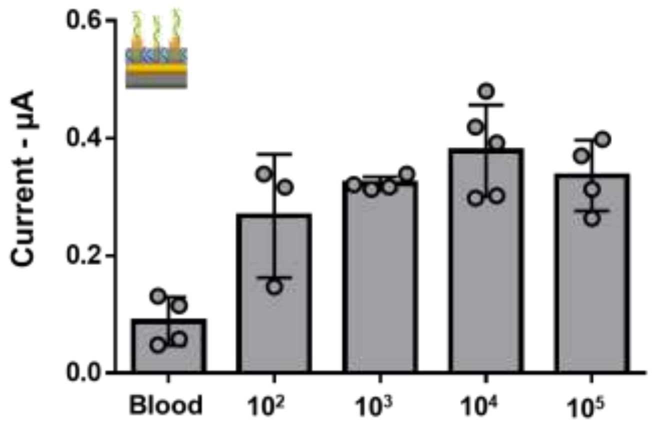

Figure S11 - Varying concentrations of *E. coli* CIM spiked in healthy blood culture samples to evaluate the response of the Liquid NanoBiosensors following incubation for 30 min at room temperature with the reaction vial mixture, and 30 min incubation on the detection chip at 37 °C.

The blood culture samples were not diluted prior to testing. However, the sample processing resulted in the overall dilution of 50%.

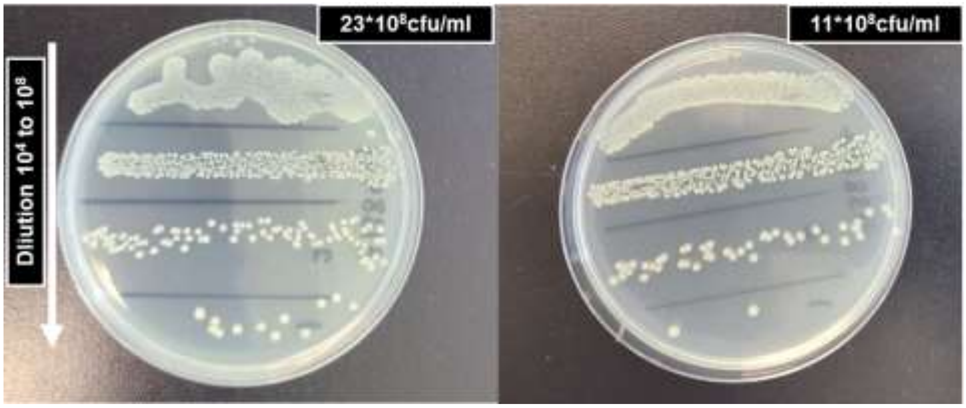

Figure S12 - E. coli+ blood culture samples cultured on LB-Agar for quantification.

Note S2 - The *E. coli*+ blood culture samples were plated on LB-Agar for quantification. All samples showed 10^8 CFU/mL range. The range of blood stream bacteria can be 10-10^4 CFU/mL, [3] or even higher ranges of 10^8 CFU/mL. [4] Bacterial growth can occur from the timepoint that blood is collected from patients to the timepoint that blood cultures are positively flagged and transferred to our research lab.

Table S1 – Summary of the oligonucleotide sequences used.

Sequence	Note
**5' TAG CTA GGA AGA GTC ACA CA-Thiol**	Capture probe
**3' – T CCT TCT CAG TGT GT – 5'**	Protecting strand

**5'- Amino - TTTTTTGTGTGACTCTTCCTAGCTrATGGTTCGATCAAGA GATGTGCGTCTTGATCGAGACCTGCGACCGTTTTTTTTTT- biotin -3'**	*E. coli* specific DNAzyme without MB tagging

References

[1] S. M. Traynor, G. A. Wang, R. Pandey, F. Li, L. Soleymani, *Angew. Chemie* **2020**, *132*, 22806.

[2] R. Pandey, D. Chang, M. Smieja, T. Hoare, Y. Li, L. Soleymani, *Nat. Chem.* **2021**, *13*, 895.

[3] E. M. K. Kurundu Hewage, D. Spear, T. M. Umstead, S. Hu, M. Wang, P. K. Wong, Z. C. Chroneos, E. S. Halstead, N. J. Thomas, *SLAS Technol. Transl. Life Sci. Innov.* **2017**, *22*, 616.

[4] J. Gao, L. Jeffries, K. E. Mach, D. W. Craft, N. J. Thomas, V. Gau, J. C. Liao, P. K. Wong, *SLAS Technol. Transl. Life Sci. Innov.* **2017**, *22*, 466.
